# Time-Dependent Density Functional Computations of the Spectrochemical Properties of Dithiolodithiole and Thiophene Electrochromic Systems

**DOI:** 10.3390/ma10090981

**Published:** 2017-08-23

**Authors:** Bruna Clara De Simone, Gloria Mazzone, Tiziana Marino, Nino Russo, Marirosa Toscano

**Affiliations:** Dipartimento di Chimica e Tecnologie Chimiche, Università della Calabria, I-87036 Arcavacata di Rende, Italy; bruna.desimone@unical.it (B.C.D.S.); tmarino@unical.it (T.M.); marirosa.toscano@unical.it (M.T.)

**Keywords:** TDDFT, organic electrochromic systems, dithiolodithiole, thiophene

## Abstract

The importance of organic electrochromic materials has grown considerably in recent decades due to their application in smart window, automotive, and aircraft technologies. Theoretical prediction of the optical properties should contribute to their better characterization and help the explanation of the experimental data. By using various exchange–correlation functionals, we show how density functional theory (DFT) and the related time-dependent formulation (TDDFT) are able to correctly reproduce the spectrochemical properties of dithiolodithiole and thiophene organic electrochromic systems.

## 1. Introduction

The electrochromism is the phenomenon associated to a persistent but reversible optical change produced by an electrochemically induced oxidation–reduction reaction [1,2,3]. The color change is achieved by a small electric current at low dc potentials on the order of a fraction of volts to a few volts. This voltage causes the addition or extraction of electrons (reduction or oxidation) with consequent modification of the spectral properties of the material. Once the potential is removed, the material remains in the new state until a competitive reaction takes place.

There are several types of electrochromic materials (EC), classified according to the characteristic absorption of their electronic states. The first type includes those compounds that, depending on their redox state, are colorless and colored, and that, for such properties, find application in the realization of transmission/absorption devices, such as smart windows [3,4] and optical filters [5]. A second class is instead characterized by materials with two redox states both absorbing in the visible spectrum region and thus presenting a change between two different colors. These materials can be used in devices such as displays [6]. Finally, there is a third class in which there are more than two states with different absorption spectra and electronically accessible, generally called ‘multicolor electrochromic materials’ [7].

The phenomenon of electrochromism is displayed in several inorganic and organic compounds, including some polymers. Electrochromic materials, depending on their intrinsic nature, can be divided into two categories: inorganic compounds, oxides of many transition metals [8]; and organic compounds. Among the inorganic compounds, the most widely used system is tungsten trioxide [8]. While bipyridine is the most studied organic compound [9] characterized to be usually colorless but that assumes an intense blue color after a reduction.

Since electrochromic devices in the last few years have aroused an increasing interest in various fields of industry (automotive, building, aerospace, etc.) [5,10], new EC molecules are continuously being designed and then synthesized. Of particular interest for their possible broad range of applications are the organic materials obtained upon assembling π-conjugated molecules from a limited number of building blocks [11].

**Scheme 1:**
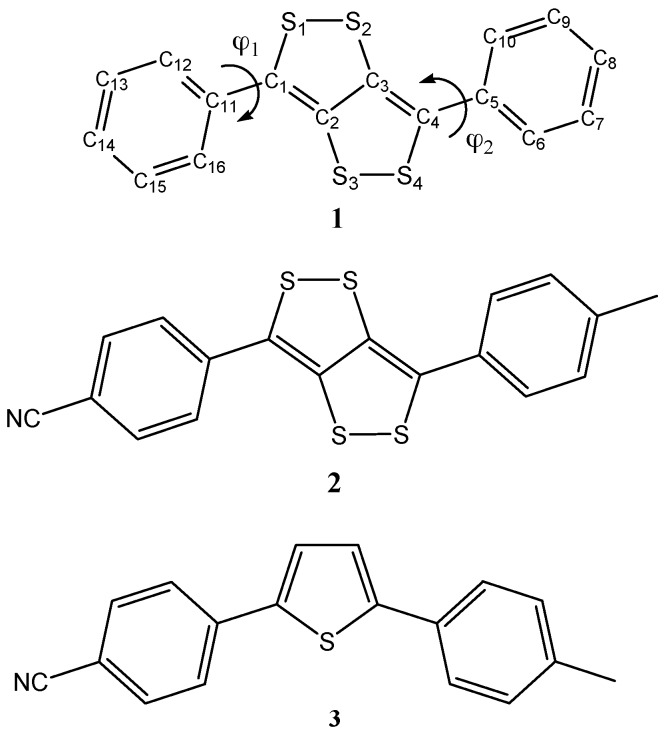
Schematic representation of the investigated compounds **1**–**3**.

Recently, a series of dithiolodithiole (C_4_S_4_) systems have been synthetized and characterized [12]. These molecules show interesting electrochemical and spectroelectrochemical properties compared to the analogous and extensively used thiophene derivatives. The C_4_S_4_ block has a 12 e- π system in the ground state and a non-aromatic nature in the quinoidal excited state that dictates their peculiar photo/electrochemical properties. The great change in UV–Vis absorption spectra upon a moderately easy oxidation process, suggests them as possible and efficient electrochromic systems. Despite their X-ray, UV–Vis, and cyclic voltammetry characterization [12], a detailed atomistic explanation of their properties has not yet been done. This can provide interesting insights into the oxidation process that could occur on the π systems or on the sulfur atoms. For this reason, a detailed theoretical investigation on the optical and electrochemical properties of the three thio-based compounds reported in Scheme 1, 3,6-Diphenyl-[1,2]dithiolo[4,3-c][1,2]dithiole (**1**), 4-(6-(4-Methylphenyl)-[1,2]dithiolo[4,3-c][1,2]dithiol-3-yl)benzonitrile (**2**) and its thiophene analogue 4-(5-(4-Methylphenyl)thiophen-2-yl)benzonitrile (**3**) has been undertaken, by using density functional-based methods.

The choice of the best computational protocol to explore the spectrochemical behavior of these newly synthesized dithiolodithiole electrochromic systems was preceded by a benchmark using different exchange and correlation functionals (XC). The selected protocol results to be able to correctly reproduce structures, excitation energies, and oxidation potentials. Moreover, the obtained results show how the phenyl rings bound to the thio-unit play an important role not only in increasing the absorption wavelengths but also in the stabilization of the radical cation species thanks to the distribution of the spin density on the entire molecule.

## 2. Computational Details

Geometries optimization in condensed phase have been performed, without any constraint, employing the 6-31G* basis set and by using the hybrid B3LYP [13,14], PBE0 [15,16], meta-hybrid M06 [17], and the range-separated hybrid wB97XD [18,19] exchange–correlation functionals as implemented in Gaussian 09 code [20]. B3LYP computations have been redone, including the D3 parametrization for the dispersion forces (B3LYP-D3) [21]. All the optimized structures have been characterized as minima by performing frequencies calculation at each level of theory just listed. Radical cation species have been treated with the unrestricted Kohn–Sham formalism. No spin contamination has been found for the open shell system being the 〈S^2^〉 value equal to 0.77.

The lowest 20 vertical excitation energies (obtained by employing the 6-31+G* basis set) have been calculated by time-dependent density functional linear response theory (TDDFT) on the previously optimized geometries. Solvent effects have been evaluated by using the non-equilibrium implementation [22] of the polarizable continuum model [23] on the previously optimized geometries. We have considered dichloromethane (ε = 8.93) as the solvent for which the experimental UV–Vis spectra and oxidation potentials are available [12].

Redox potentials computed with respect to ferrocene (E (Fc + /Fc) = 4.58 V at M06/6-31G* level of theory) have been obtained according to the Nernst equation [24]:

(1) $$E=-\frac{\Delta {\left({\Delta G}_{s}\right)}^{\mathrm{redox}}}{nF}$$

where *F* is the Faraday constant, *n* is the number of electrons transferred in the reaction, and$\;\Delta {\left({\Delta G}_{s}\right)}^{\mathrm{redox}}$ is

(2) $$\Delta {\left({\Delta G}_{s}\right)}_{redox}^{I}={\Delta G}_{s}\left(\mathrm{Compd}\right)-{\Delta G}_{s}\left({\mathrm{Compd}}^{∔}\right)$$

(3) $$\Delta {\left({\Delta G}_{s}\right)}_{redox}^{II}={\Delta G}_{s}\left(\mathrm{Compd}\right)-{\Delta G}_{s}\left({\mathrm{Compd}}^{2+}\right)$$

for $E_{ox}^{I}$ and $E_{ox}^{II}$, respectively.

A similar computational protocol has been recently and successfully used for the determination of a series of electronic and spectroscopic properties, including the spectrochemical ones [25,26,27,28,29].

## 3. Results and Discussion

**Figure 1:**
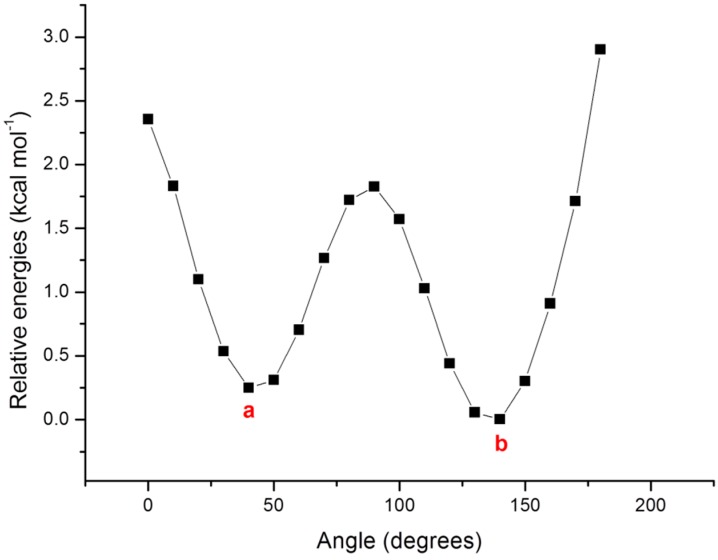
Potential energy surface scan of the torsion angle φ_2_.

As a first step of the work we have performed a conformational analysis on compound **1** (see Scheme 1) for which the X-ray data are available [12]. Since the system possesses two torsional angles, a preliminary scan on φ_2_ angle of compound **1** (see Scheme 1) has been performed. A potential energy surface scan of Figure 1 shows two minima (**a** and **b**) along the potential energy profile. The absolute minimum (**b**) has been found at a value of 140 degrees, while the other one (**a**) lies about 0.3 kcal mol^−1^ above and is characterized by a torsional angle of 40 degrees.

The interconversion between the two minima requires a low barrier (about 2 kcal mol^−1^), then, in principle, both can be populated. Performing the fully optimization of the two minima, the energy difference between them becomes smaller falling in the range of 0.21 to 0.29 kcal mol^−1^ depending on the used exchange–correlation functional (XC).

The main geometrical parameters for **a** and **b**, obtained employing the different XC, are reported in Table 1. Comparison with the X-ray results shows as all the employed XC functionals give bond lengths and valence angle values in excellent agreement with the experimental counterparts [12]. The main difference concerns the dihedral angles φ_1_ that deviates by about 12° with respect to the X-ray values. However, deviations can be explained taking into account the fact that in solid state the crystalline environment imposes a different rearrangement.

In order to verify if the different dihedral angles characterizing the two minima can affect the excitation energies, TDDFT computations have been done for both the conformations. Results are reported in Table 2. From this table, it appears evident that the two minima give very similar absorption energies being the difference of few nm. In particular, the better agreement with the absorption maxima derived by UV–Vis spectrum (458 nm) [12] is found in the case of absolute minimum **b**. All the employed XCs, with the exception of wB97XD, which provides a value very far from experimental one, slightly overestimate the excitation energies with an average error of 18 nm. From the comparison of both structural and electronic properties computed for molecule **1** with the experimental results, a quite satisfactory agreement emerges. Therefore, the used protocol can be reliably applied to the study of compounds **2** and **3**.

The vertical transition energies with the relative oscillator strength and the main molecular orbitals (MO) involved in the transitions for compounds **2** and **3** are reported in Table 3. As regards to compound **2**, it is noteworthy that for the neutral system the computed B3LYP, B3LYP-D3, M06, and PBE0 excitation energies are in very good agreement with the corresponding experimental ones. While wB97XD gives values rather far from experimental data, as found for other EC materials [25,27]. Therefore, with the exception of the data coming from wB97XD computations, the excitation energies computed with the other XC functionals show good agreement with experimental data. Examining the average error on the absorption wavelength computed by using all the above mentioned XC functionals for all the considered species of compound **2**, that are neutral, radical cation, and dication species, B3LYP-D3 and M06 return the best agreement. For this reason, for compound **3** only these functionals have been used and unless otherwise noted, in what follows, only the results obtained with the M06 functional will be discussed.

**Figure 2:**
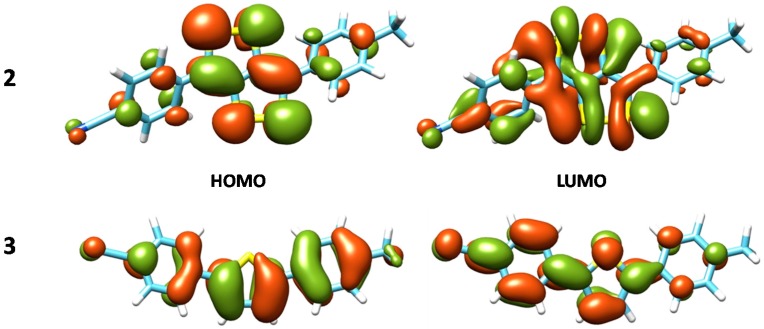
Graphical representation of HOMO and LUMO MOs for the neutral compound **2** and **3** at the M06/6-31G* level of theory.

Compound **2** in its neutral form present an intense absorption band at 543 nm (vs. 517 nm experimentally determined [12]) which corresponds to the HOMO–LUMO transition and is π-π* in nature (for the orbital composition see Figure 2).

**Figure 3:**
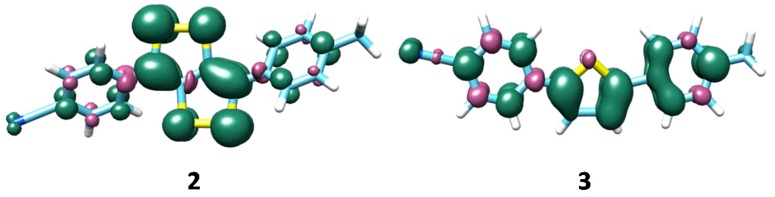
M06/6-31G* spin density contour plot for radical cations of compounds **2** and **3**.

In the **2^+∙^** radical cation the experimental Q band, for which only an estimate λ_max_ exists, is composed by two transitions, with almost the same oscillator strength, that lies at 640 nm and 620 nm. These two peaks are mainly due to transitions involving HOMO(β)→LUMO(β) and HOMO(α)→LUMO(α) orbitals. The transition at 400 nm involves the inner HOMO-5(β) and the LUMO(β) orbitals. It was previously hypothesized that, in the oxidation process of this molecule, the π electrons of phenyl rings other than the sulfur atoms are involved [12]. The analysis of the spin density distribution reported in Figure 3 clearly evidences the role of the phenyl rings in delocalizing the spin density and consequently in the stabilization of the radical cation.

The dicationic species **2^2+^** shows only a band resulting from two electronic transitions: HOMO→LUMO (613 nm) and HOMO-2→LUMO (498 nm). The experimental absorption measurements indicate a broad band red shifted with respect to the corresponding band of neutral species (the estimated experimental λ_max_ is 570 nm [12]).

**Figure 4:**
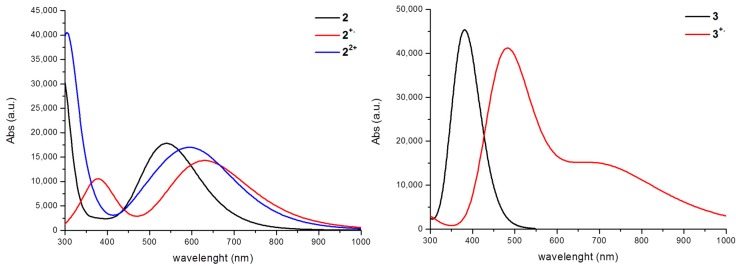
M06/6-31+G* computed absorption spectra for neutral and cationic species of compounds **2** and **3**.

The sequential oxidation of **2** produces interesting absorption changes, as evidenced in Figure 4, that are well predicted by theoretical computations (see Table 3). The formation of the **2^+∙^** radical cation species gives rise to a new band at 381 nm and induces a red shift of the band from 543 nm to 640 nm. A further oxidation to **2^2+^** produces a blue shift of 27 nm of the Q band with respect to that observed for the radical cation.

The excitation energies computed for the neutral species **3** (see Table 3) evidence an UV band at 381 nm (387 nm) at M06 (B3LYP-D3) that is in good agreement with the maximum experimental band at about 365 nm [12]. The transition can be classified as π→π* and involves HOMO and LUMO orbitals (see Figure 2 and Table 3). The oxidation of this system gives a radical cation with the UV band red-shifted at 483 nm and a new band in the near-IR region at 721 nm. This new band is totally due to a HOMO (π)→LUMO (π) transition.

The spin density distribution map (Figure 3) shows a low density on the sulfur atom and a substantial one on the entire π system (thiophene and phenyl rings), contrariwise to **2^+∙^**, for which a marked contribution of sulfur atoms has been found. This can be explained on the basis of structural features. Indeed, the different distribution found in radical cations of **2** and **3** can be related to the orientation of phenyl rings with respect to the thio unit in the two cases. In the first one, methyl phenyl and nitrile phenyl are orientated at 142 and 141 degrees, respectively with respect to the thio core, while 1 and 7 degrees are the deviations from the thio unit plane of the correspondent rings found in compound **3**. As is known, the planarity between π systems maximizes the conjugation and then the electronic delocalization, so the deviation from the planarity more pronounced in compound **2** than in compound **3** (e.g., looking at φ_2_ = 38 vs. 1 degrees) could be the explanation why the phenyl rings are more involved in radical cation of compound **3** than in that of compound **2**.

The HOMO–LUMO energy gap values, computed at M06 level of theory, of 4.09 and 2.99 eV for **2** and **3**, respectively are overestimated by about 0.8 eV with respect to those determined experimentally (3.10 eV and 2.14 eV for **2** and **3**, respectively) [12]. However, considering that the energy gaps computed at the M06 level for the two molecules differ of 1.10 eV, the agreement results much better, being 0.96 eV that measured [12]. As expected, an increase of the HOMO–LUMO energy gap results in a decrease of the absorption wavelengths values. As expected, an increase of the HOMO–LUMO energy gap results in a decrease of the absorption wavelengths values.

The obtained M06 oxidation potentials, calculated according to Equation (1), are reported in Table 4 together with the available experimental counterparts.

The agreement between theory and experiment is reasonable although the computed potentials for the first oxidation of **2** and **3** are overestimated by 0.18 and 0.15 V with respect to the experimental counterparts. In any case, the trend for the first and second oxidation potential of **2** is well reproduced and the highest potential for **3** is confirmed.

## 4. Conclusions

An accurate theoretical computation of the structural and electronic properties of some newly synthetized dithiolodithiole (C_4_S_4_) electrochromic systems, performed by using different exchange–correlation functionals in the framework of density functional theory has been carried out. The obtained results show how the used computational protocol is able to correctly reproduce structures, excitation energies, oxidation potentials, and spectrochemical behavior of these interesting organic materials. Indeed, all the employed exchange–correlation functionals are able to give accurate geometrical parameters in good agreement with the experimental data. The slight differences found can be attributed to the different environment used in the experimental and theoretical determinations (solid versus liquid phases). In particular, the meta-hybrid M06 and the hybrid B3LYP, B3LYP-D3, PBE0 exchange–correlation functionals give vertical excitation energies close to the measured counterpart for neutral and charged species, allowing to correctly reproduce the behavior of UV–Vis spectrochemical measurements. Also, the computed oxidation potentials can be considered reliable due to their satisfactory agreement with the measured ones.

Furthermore, from the computations it can be seen that the presence and nature of the terminal phenyl rings play an important role not only in increasing absorption wavelengths, but also in the stabilization of the radical cation species that are characterized by the distribution of the spin density on the entire molecule.

## Figures and Tables

**Table 1:** Main structural parameters for the two minima **a** and **b**, intercepted along the potential energy surface scan of φ_2_ dihedral angle in compound **1**, obtained employing different exchange–correlation functionals in dichloromethane environment. Distances are in Å. Valence and torsional angles are in degrees. Values for the relative minimum **a** are in parentheses.

Parameter	X-ray ^a^	B3LYP	B3LYPD3	M06	PBE0	wB97XD
C_1_-C_2_	1.365	1.361	1.359	1.357	1.359	1.347
		(1.360)	(1.359)	(1.357)	(1.358)	(1.347)
C_2_-C_3_	1.444	1.448	1.449	1.442	1.442	1.453
		(1.449)	(1.450)	(1.442)	(1.443)	(1.453)
C_2_-S_3_	1.757	1.786	1.788	1.774	1.767	1.773
		(1.787)	(1.788)	(1.775)	(1.767)	(1.773)
S_3_-S_4_	2.084	2.132	2.134	2.123	2.102	2.108
		(2.134)	(2.137)	(2.125)	(2.104)	(2.110)
C_1_-C_2_-S_3_	126.5	125.4	125.4	125.6	125.9	126.0
		(125.4)	(125.4)	(125.7)	(126.0)	(126.1)
C_3_-C_2_-S_3_	113.4	113.6	113.7	113.7	(113.7	113.7
		(113.6)	(113.7)	(113.7)	(113.7)	(113.7)
C_2_-S_3_-S_4_	95.8	95.0	94.9	95.0	95.3	95.1
		(94.9)	(94.8)	(94.9)	(95.2)	(95.0)
C_2_-C_3_-C_4_	120.1	120.7	120.6	120.3	120.1	120.0
		(120.7)	(120.6)	(120.3)	(120.1)	(120.0)
φ_1_	154.6	141.3	136.5	143.3	143.5	134.7
		(140.7)	(136.0)	(143.1)	(143.2)	(134.3)
φ_2_	26.1	141.0	136.6	142.3	143.2	136.1
		(38.6)	(43.3)	(36.0)	(35.9)	(44.6)

^a^ Ref. [12].

**Table 2:** Vertical excitation energies, ΔE in eV, absorption wavelengths, λ_theo_ in nm, and oscillator strength, *f*, computed in dichloromethane solvent and employing different exchange–correlation functionals for the two minima of compound **1**.

Conformer	Theory	λ_theo_/nm	ΔE/eV	*f*
**1a**	B3LYP	492	2.52	0.309
	B3LYP_D3	489	2.53	0.259
	M06	492	2.52	0.346
	PBE0	480	2.58	0.366
	wB97XD	402	3.08	0.321
**1b**	B3LYP	480	2.58	0.405
	B3LYP_D3	474	2.62	0.369
	M06	479	2.59	0.424
	PBE0	471	2.63	0.380
	wB97XD	399	3.10	0.470
	Exp ^a^	458		

^a^ Ref. [12].

**Table 3:** Vertical excitation energies, ΔE in eV, absorption wavelengths, λ_theo_ in nm, and oscillator strength, *f*, computed in dichloromethane for systems **2** and **3** employing different exchange–correlation functionals. The main Molecular Orbital contributions (%) for each absorption band computed at B3LYP/6-31+G* are provided.

Compound	XC	ΔE	λ_theo_	*f*	*MO contribution* ^a,b^	λ_exp_^c^
**2**	B3LYP	2.24	554	0.412	H→L, 96%	517
	B3LYP-D3	2.24	554	0.354		
	M06	2.28	543	0.405		
	PBE0	2.31	537	0.457		
	wB97XD	2.91	425	0.460		
**2^+∙^**	B3LYP	1.91	648	0.173	H(β) ^c^→L(β), 100%	≈650
		2.00	619	0.146	H(α)→L(α), 91%	
		3.12	397	0.130	H-5 (β)→L (β), 92%	≈400
	B3LYP-D3	1.92	646	0.164		
		2.02	613	0.144		
		3.10	400	0.137		
	M06	1.94	640	0.220		
		2.00	620	0.128		
		3.25	381	0.143		
	PBE0	2.00	619	0.280		
		2.02	606	0.067		
		3.29	376	0.134		
	wB97XD	2.25	551	0.242		
		2.55	550	0.132		
		3.32	374	0.185		
**2^2+^**	B3LYP	1.92	644	0.366	H→L, 99%	≈570
		2.32	533	0.149	H-2→L, 88%	
	B3LYP-D3	1.91	649	0.336		
		2.31	536	0.145		
	M06	2.02	613	0.371		
		2.49	498	0.154		
	PBE0	2.04	606	0.370		
		2.48	499	0.155		
	wB97XD	2.43	509	0.544		
		3.02	411	0.107		
**3**	B3LYP-D3	3.20	387	1.090	H→L,99%	≈360
	M06	3.25	381	1.120		
**3^+∙^**	B3LYP-D3	1.68	738	0.163	H(β)→L(β), 100%	
		1.75	709	0.157	H(β)→L(β), 100%	
		2.59	478	1.033	H(α)→L(α), 100%	
	M06	1.72	721	0.278		
		2.57	483	0.966		

^a^ H and L stand for HOMO and LUMO orbitals; ^b^ for the open shell systems, α and β refer to the electrons; ^c^ ref [12].

**Table 4:** Computed oxidation potentials for **2** and **3** in dichloromethane solvent, employing M06 exchange–correlation functional

Compd.	E_ox_^I^/V		E_ox_^II^/V	
	Computed	Experimental	Computed	Experimental
**2**	0.31	0.13	0.88	0.71
**3**	0.85	≈1.00	-	-
